# Supplementary material for: Characterizing a psychiatric symptom dimension related to deficits in goal-directed control
Source: eLife. 2016 Mar 1;5:e11305. doi: 10.7554/eLife.11305 (PMC4786435; doi:10.7554/eLife.11305)
Supplement: Supplementary file 1. — a For the purposes of comparison across studies using the EAT, responses on the 6-point scale are converted as follows: (1:0, 2:0, 3:0, 4:1, 5:2, 6:3) and totaled to produce the mean reported above (Everitt and Robbins, 2005). For analysis purposes (including correlations reported above), however, the continuous (i.e. 1,2,3,4,5,6) values were used (M=33.31, SD=17.36). Likewise, for LSAS, the mean reported above corresponds to the summed total of the full questionnaire - i.e. including answers to both avoidance and fear/anxiety probes. However, for the purposes of analyses, we used the average of the avoidance and fear/anxiety answers for each item. b Positive t-values indicate higher scores among males, while negative t-values indicate higher scores in females. (B) Results from Basic Logistic Regression Model in Experiment 1 (N=548) and Experiment 2 (N=1413) with Age, Gender and IQ as fixed effects predictors. *p<0.05 ** p<0.01 ***p<0.001SE=standard error. (C) Questionnaire Total Scores and Model-Free Learning*p<0.05 ** p<0.01 ***p<0.001SE=standard error. Each row reflects the results from an independent analysis where each questionnaire total score (z-transformed) was entered as SymptomScorez in the following model: glmer(Stay ~ Reward * Transition * SymptomScorez + Reward * Transition * (IQz + Agez + Gender) + (Reward * Transition + 1 | Subject)). Model-free statistics refer to the following interaction: SymptomScorez x Reward. For each, positive β values indicate that the symptom score is associated with greater model-free learning, while negative β values indicate that the symptom score is associated with reduced model-free learning. DOI: http://dx.doi.org/10.7554/eLife.11305.011 [file elife-11305-supp1.docx]

**Supplementary File 1A. Questionnaire Total Scores in Experiments 1 and 2**

|  | **Mean (SD)** | **Age** | | **Gender ^b^** | | | **IQ** | |
| --- | --- | --- | --- | --- | --- | --- | --- | --- |
| **Experiment 1** |  | **r** | ***p*** | **t** | ***p*** | | **r** | ***p*** |
| OCD (OCI-r) | 15.69 (11.28) | **-.20** | **<.001** | -1.10 | .271 | | -.00 | .970 |
| Depression (SDS) | 39.23 (11.5) | **-.17** | **<.001** | -1.67 | 096 | | -.02 | .656 |
| Trait Anxiety (STAI-T) | 42.64(13.39) | **-.19** | **<.001** | -1.05 | .296 | | .02 | .705 |
|  |  |  |  |  |  | |  |  |
| **Experiment 2** |  |  |  |  |  | |  |  |
| OCD (OCI-r) | 14.4 (11.59) | **-.21** | **<.001** | 0.68 | .496 | | -.04 | .131 |
| Depression (SDS) | 39.57(10.99) | **-.14** | **<.001** | **-3.39** | **<.001** | | -.02 | .553 |
| Trait Anxiety (STAI-T) | 43.07(13.58) | **-.18** | **<.001** | -1.98 | .047 | | .03 | .303 |
| Eating Disorders (EAT-26) | 8.34(8.64) ^a^ | **-.08** | **.002** | **-5.54** | **<.001** | | .00 | .885 |
| Impulsivity (BIS-10) | 61.06 (11.2) | **-.12** | **<.001** | 1.80 | .072 | | -.05 | .064 |
| Alcohol Addiction (AUDIT) | 4.67 (5.47) | **-.14** | **<.001** | **6.72** | **<.001** | | **.07** | **.013** |
| Social Anxiety (LSAS) | 44.15 (28.92) | **-.06** | **.02** | **-5.59** | **<.001** | | .01 | .577 |
| Apathy (AES) | 31.51 (8.19) | **-.08** | **.004** | **3.02** | **.002** | | .07 | .006 |
| Schizotypy (SSMS) | 13.47 (7.78) | **-.15** | **<.001** | **-1.99** | **.046** | | .01 | .596 |
|  |  | |  | | |  |  |  |

**^a^ For the purposes of comparison across studies using the EAT, responses on the 6-point scale are converted as follows: (1:0, 2:0, 3:0, 4:1, 5:2, 6:3) and totaled to produce the mean reported above^1^. For analysis purposes (including correlations reported above), however, the continuous (i.e. 1,2,3,4,5,6) values were used (M=33.31, SD=17.36). Likewise, for LSAS, the mean reported above corresponds to the summed total of the full questionnaire - i.e. including answers to both avoidance and fear/anxiety probes. However, for the purposes of analyses, we used the average of the avoidance and fear/anxiety answers for each item.**

**^b^ Positive t-values indicate higher scores among males, while negative t-values indicate higher scores in females.**

**Supplementary File 1B: Results from Basic Logistic Regression Model in Experiment 1 (N=548) and Experiment 2 (N=1413) with Age, Gender and IQ as fixed effects predictors.**

|  | ***Experiment 1 (N=548)*** | | | ***Experiment 2 (N=1413)*** | | |
| --- | --- | --- | --- | --- | --- | --- |
| **Coefficient** | **β (SE)** | ***z*-value** | ***p*-value** | **β (SE)** | ***z*-value** | ***p*-value** |
| **(Intercept)** | **1.951(0.05)** | **38.87** | **<.001 ***** | **1.752(0.03)** | **61.59** | **<.001 ***** |
| **Reward** | **0.711(0.03)** | **23.49** | **<.001 ***** | **0.583(0.02)** | **35.69** | **<.001 ***** |
| **Transition** | **0.073(0.02)** | **4.46** | **<.001 ***** | **0.062(0.01)** | **6.93** | **<.001 ***** |
| **IQ** | **0.100(0.05)** | **2.07** | **0.038 *** | **0.196(0.03)** | **6.90** | **<.001 ***** |
| **Age** | **0.249(0.05)** | **5.15** | **<.001 ***** | **0.180(0.03)** | **6.38** | **<.001 ***** |
| Gender | -0.055(0.05) | -1.10 | 0.272 | -0.025(0.03) | -0.89 | 0.372 |
| **Reward:Transition** | **0.287(0.02)** | **13.07** | **<.001 ***** | **0.266(0.01)** | **22.33** | **<.001 ***** |
| Reward:IQ | 0.005(0.03) | 0.19 | 0.848 | -0.007(0.02) | -0.41 | 0.679 |
| **Transition:IQ** | **0.031(0.01)** | **2.24** | **0.025 *** | **0.022(0.01)** | **2.78** | **0.005 **** |
| **Reward:Age** | **0.127(0.03)** | **4.38** | **<.001 ***** | **0.117(0.02)** | **7.33** | **<.001 ***** |
| Transition:Age | -0.021(0.02) | -1.44 | 0.150 | 0.006(0.02) | 0.69 | 0.488 |
| **Reward:Gender** | -0.043(0.03) | -1.47 | 0.143 | **-0.044(0.02)** | **-2.72** | **0.006 **** |
| Transition:Gender | 0.027(0.01) | 1.90 | 0.058 | -0.001(0.00) | -0.16 | 0.873 |
| **Reward:Transition:IQ** | **0.099(0.02)** | **4.90** | **<.001 ***** | **0.102(0.01)** | **9.01** | **<.001 ***** |
| **Reward:Transition:Age** | **-0.041(0.02)** | **-1.97** | **0.049 *** | **-0.037(0.01)** | **-3.26** | **0.001 **** |
| **Reward:Transition:Gender** | 0.019(0.02) | 0.91 | 0.362 | **0.028(0.01)** | **2.48** | **0.013 *** |

****p*<.05 ** *p*<.01 ****p*<.001**

**SE=standard error**

**Supplementary File 1C. Questionnaire Total Scores and Model-Free Learning**

| **Questionnaire** | **β (SE)** | ***z*-value** | ***p*-value** |
| --- | --- | --- | --- |
| Experiment 1 (N=548) |  |  |  |
| OCD (OCI-R) | 0.030(0.03) | 1.03 | .303 |
| Depression (SDS) | -0.044(0.03) | -1.53 | .127 |
| Trait Anxiety (STAI-T) | -0.022(0.03) | -0.75 | .452 |
| Experiment 2 (N=1413) |  |  |  |
| Eating Disorders (EAT-26) | -0.017(0.02) | -1.05 | .294 |
| Impulsivity (BIS) | -0.010(0.02) | -0.64 | .525 |
| OCD (OCI-R) | -0.012(0.02) | -0.73 | .467 |
| Alcohol Addiction (AUDIT) | -0.013(0.02) | 0.78 | .433 |
| Schizotypy (SCZ) | -0.019(0.02) | -1.17 | .240 |
| Depression (SDS) | -0.007(0.02) | -0.46 | .646 |
| Trait Anxiety (STAI-T) | -0.005(0.02) | -0.33 | .739 |
| Apathy (AES) | -0.021(0.02) | -1.35 | .177 |
| Social Anxiety (LSAS) | 0.004(0.02) | 0.24 | .812 |
|  |  |  |  |

****p*<.05 ** *p*<.01 ****p*<.001**

**SE=standard error**

**Each row reflects the results from an independent analysis where each questionnaire total score (z-transformed) was entered as SymptomScorez in the following model: glmer(Stay ~ Reward * Transition * SymptomScorez + Reward * Transition * (IQz + Agez + Gender) + (Reward * Transition + 1 | Subject)). Model-free statistics refer to the following interaction: SymptomScorez x Reward. For each, positive β values indicate that the symptom score is associated with greater model-free learning, while negative β values indicate that the symptom score is associated with reduced model-free learning.**

**Supplementary References**

1 Garner, D. M., Olmsted, M. P., Bohr, Y. & Garfinkel, P. E. The eating attitudes test: psychometric features and clinical correlates. *Psychol Med* **12**, 871-878 (1982).
