# Supplementary material for: Characterizing a psychiatric symptom dimension related to deficits in goal-directed control
Source: eLife. 2016 Mar 1;5:e11305. doi: 10.7554/eLife.11305 (PMC4786435; doi:10.7554/eLife.11305)
Supplement: Supplementary file 2. — Summary of item loadings onto ‘Anxious-Depression’ factor. The top loading items from each questionnaire are displayed in descending order, provided they are above a threshold loading of +/- 0.25. Words in parentheses, e.g. “(do not)” are added here (but were not presented to participants) to facilitate interpretation of the direction of effects for items that are reverse-coded. (B) ‘Top loading items on ‘Compulsive Behavior and Intrusive Thought’. Summary of item loadings onto ‘Compulsive Behavior and Intrusive Thought’ factor. The top loading items from each questionnaire are displayed in descending order, provided they are above a threshold loading of +/- 0.25. (C) ‘Top loading items on ‘Social Withdrawal’ factorSummary of item loadings onto ‘Social Withdrawal’ factor. The top loading items from each questionnaire are displayed in descending order, provided they are above a threshold loading of +/- 0.25. Words in parentheses, e.g. '(do not)' are added here (but were not presented to participants) to facilitate interpretation of the direction of effects for items that are reverse-coded. DOI: http://dx.doi.org/10.7554/eLife.11305.012 [file elife-11305-supp2.docx]

**Supplementary File 2A. ‘Top loading items on ‘Anxious-Depression’ factor**

| **Factor 1: Anxious-Depression** | | | |
| --- | --- | --- | --- |
| **Questionnaire** | **Item** |  | **Loading** |
| STAI-T | I am (not) happy |  | 0.779 |
| STAI-T | I (do not) feel satisfied with self |  | 0.746 |
| AES | I (do not) have motivation |  | 0.742 |
| STAI-T | I am (not) content |  | 0.73 |
| SDS | I (do not) feel that I am useful and needed. |  | 0.722 |
| SDS | My life is (not) pretty full. |  | 0.71 |
| AES | I (do not) have initiative |  | 0.709 |
| STAI-T | I (do not) feel pleasant |  | 0.705 |
| STAI-T | I (do not) feel secure |  | 0.675 |
| SDS | I (do not) feel hopeful about the future. |  | 0.667 |
| STAI-T | I feel like failure |  | 0.663 |
| AES | I (do not) get things done during the day |  | 0.625 |
| STAI-T | I (not) am a steady person |  | 0.621 |
| SDS | I (do not) still enjoy the things I used to do. |  | 0.605 |
| SDS | I feel down-hearted and blue |  | 0.595 |
| STAI-T | I feel inadequate |  | 0.589 |
| SDS | I (do not) find it easy to do the things I used to. |  | 0.56 |
| AES | Getting things done during the day is (not) important to me |  | 0.557 |
| AES | I (do not) approach life with intensity |  | 0.549 |
| SDS | I (do not) find it easy to make decisions. |  | 0.548 |
| AES | I am (not) interested in things |  | 0.545 |
| BIS | I (do not) plan for job security. |  | 0.543 |
| STAI-T | I feel that difficulties are piling up so that I can't overcome them |  | 0.54 |
| STAI-T | I am (not) cool, calm and collected |  | 0.529 |
| AES | Seeing a job through to the end is (not) important |  | 0.506 |
| BIS | I do not concentrate easily. |  | 0.505 |
| BIS | I am (not) self-controlled. |  | 0.495 |
| BIS | I (do not) plan tasks carefully. |  | 0.485 |
| STAI-T | I (do not) make decisions easily |  | 0.475 |
| STAI-T | I lack self-confidence |  | 0.473 |
| SDS | My mind is (not) as clear as it used to be. |  | 0.461 |
| STAI-T | I wish I could be as happy as others seem to be |  | 0.46 |
| AES | I put little effort into anything |  | 0.454 |
| AES | I (do not) spend time doing things that interest me |  | 0.449 |
| BIS | I am (not) happy-go-lucky. |  | 0.448 |
| BIS | I am (not) future oriented. |  | 0.438 |
| BIS | I (do not) save regularly. |  | 0.436 |
| BIS | I am (not) a careful thinker. |  | 0.421 |
| STAI-T | I (do not) feel rested |  | 0.42 |
| AES | I (do not) have friends |  | 0.412 |
| AES | Getting things started on my own is (not) important to me |  | 0.412 |
| BIS | I am (not) a steady thinker. |  | 0.411 |
| STAI-T | I get in a state of tension or turmoil as I think over my recent concerns and interests |  | 0.408 |
| AES | When something good happens, I (do not) get excited |  | 0.404 |
| SSMS | Do you frequently have difficulty in starting to do things? |  | 0.399 |
| SDS | I feel that others would be better off if I were dead. |  | 0.396 |
| SDS | I am more irritable than usual. |  | 0.389 |
| SDS | I get tired for no reason. |  | 0.384 |
| BIS | I (do not) plan trips well ahead of time. |  | 0.373 |
| STAI-T | I feel nervous and restless |  | 0.371 |
| BIS | I don’t pay attention. |  | 0.367 |
| AES | I (do not) have an accurate understanding of my problems |  | 0.365 |
| SDS | I have crying spells or feel like it |  | 0.357 |
| SSMS | Are you a person whose mood goes up and down easily? |  | 0.354 |
| BIS | I spend or charge more than I earn. |  | 0.35 |
| STAI-T | I have disturbing thoughts |  | 0.342 |
| SSMS | Is it hard for you to make decisions? |  | 0.341 |
| SSMS | Have you ever felt the urge to injure yourself? |  | 0.333 |
| BIS | I do things without thinking. |  | 0.313 |
| SDS | I am restless and can’t keep still. |  | 0.305 |
| SSMS | Do you find it difficult to keep interested in the same thing for a long time? |  | 0.3 |
| SSMS | Do you often feel the impulse to spend money, which you know you can’t afford? |  | 0.298 |
| STAI-T | I take disappointments so keenly I can't put them out of my mind |  | 0.293 |
| SSMS | Do you (not) feel very close to your friends? |  | 0.284 |
| SDS | Morning is (not) when I feel best |  | 0.283 |
| SDS | I have trouble sleeping at night. |  | 0.278 |
| SSMS | Do you at times have an urge to do something harmful or shocking? |  | 0.276 |
| SSMS | Are you usually (not) in an average kind of mood, not too high and not too low? |  | 0.272 |
| BIS | I have racing thoughts. |  | 0.271 |
| BIS | I act on impulse. |  | 0.271 |
| AES | Getting together with friends is (not) important to me |  | 0.27 |
| SSMS | Are there very few things that you have ever enjoyed doing? |  | 0.269 |
| BIS | I often have extraneous thoughts when thinking. |  | 0.269 |
| EAT-26 | I (do not) display self-control around food. |  | 0.263 |
| BIS | I (do not) make-up my mind quickly. |  | 0.261 |
| SSMS | Do you often have difficulties in controlling your thoughts? |  | 0.26 |
| SDS | I (do not) still enjoy sex. |  | 0.259 |
| AES | I am less concerned about my problems than I should be |  | 0.255 |
| AES | I am (not) interested in learning new things |  | 0.253 |
|  |  |  |  |

**Summary of item loadings onto ‘Anxious-Depression’ factor. The top loading items from each questionnaire are displayed in descending order, provided they are above a threshold loading of +/- 0.25. Words in parentheses, e.g. “(do not)” are added here (but were not presented to participants) to facilitate interpretation of the direction of effects for items that are reverse-coded.**

**Supplementary File 2B. ‘Top loading items on ‘Compulsive Behavior and Intrusive Thought’**

| **Factor 2: Compulsive Behavior and Intrusive Thought** | | | |
| --- | --- | --- | --- |
| **Questionnaire** | **Item** |  | **Loading** |
| OCI-R | I sometime shave to wash or clean myself simply because I feel contaminated. |  | 0.564 |
| OCI-R | I am upset by unpleasant thoughts that come into my mind against my will. |  | 0.561 |
| OCI-R | I feel I have to repeat certain numbers. |  | 0.555 |
| OCI-R | I repeatedly check doors, windows, drawers, etc. |  | 0.555 |
| OCI-R | I frequently get nasty thoughts and have difficulty in getting rid of them. |  | 0.554 |
| OCI-R | I repeatedly check gas and water taps and light switches after turning them off. |  | 0.534 |
| OCI-R | I feel that there are good and bad numbers. |  | 0.523 |
| EAT-26 | Am preoccupied with the thought of having fat on my body. |  | 0.519 |
| EAT-26 | Have gone on eating binges where I feel that I may not be able to stop. |  | 0.514 |
| OCI-R | I wash my hands more often and longer than necessary. |  | 0.511 |
| OCI-R | I find it difficult to control my own thoughts. |  | 0.504 |
| OCI-R | I get upset if objects are not arranged properly. |  | 0.497 |
| EAT-26 | Am preoccupied with a desire to be thinner. |  | 0.495 |
| EAT-26 | Feel extremely guilty after eating. |  | 0.491 |
| OCI-R | I need things to be arranged in a particular order. |  | 0.49 |
| OCI-R | I feel compelled to count while I am doing things. |  | 0.489 |
| EAT-26 | Vomit after I have eaten. |  | 0.477 |
| OCI-R | I check things more often than necessary. |  | 0.474 |
| EAT-26 | Give too much time and thought to food. |  | 0.473 |
| OCI-R | I find it difficult to touch an object when I know it has been touched by strangers or certain people |  | 0.473 |
| EAT-26 | Have the impulse to vomit after meals. |  | 0.471 |
| EAT-26 | Find myself preoccupied with food. |  | 0.464 |
| EAT-26 | Feel that food controls my life. |  | 0.454 |
| EAT-26 | Am terrified about being overweight. |  | 0.453 |
| OCI-R | I get upset if others change the way I have arranged things. |  | 0.448 |
| BIS | I have racing thoughts. |  | 0.445 |
| EAT-26 | Avoid eating when I am hungry. |  | 0.44 |
| EAT-26 | Engage in dieting behavior. |  | 0.439 |
| STAI-T | Some unimportant thought runs through my mind and bothers me |  | 0.433 |
| OCI-R | I collect things I don’t need. |  | 0.428 |
| EAT-26 | Feel uncomfortable after eating sweets. |  | 0.425 |
| STAI-T | I have disturbing thoughts |  | 0.421 |
| EAT-26 | Like my stomach to be empty. |  | 0.41 |
| OCI-R | I avoid throwing things away because I am afraid I might need them later. |  | 0.406 |
| EAT-26 | Think about burning up calories when I exercise. |  | 0.406 |
| EAT-26 | Feel that others pressure me to eat. |  | 0.406 |
| AUDIT | How often during the last year have you had a feeling of guilt or remorse after drinking? |  | 0.399 |
| AUDIT | How often during the last year have you needed a first drink in the morning to get yourself going after a heavy drinking session? |  | 0.369 |
| BIS | I say things without thinking. |  | 0.364 |
| SSMS | Do you often have difficulties in controlling your thoughts? |  | 0.358 |
| SDS | My heart beats faster than usual. |  | 0.357 |
| OCI-R | I have saved up so many things that they get in the way. |  | 0.356 |
| AUDIT | How often during the last year have you failed to do what was normally expected of you because of drinking? |  | 0.355 |
| AUDIT | How often during the last year have you been unable to remember what happened the night before because of your drinking? |  | 0.354 |
| EAT-26 | Feel that others would prefer if I ate more. |  | 0.353 |
| SSMS | When you look in the mirror does your face sometimes seem quite different from usual? |  | 0.35 |
| BIS | I often have extraneous thoughts when thinking. |  | 0.344 |
| EAT-26 | Eat diet foods. |  | 0.34 |
| STAI-T | I worry too much over something that really doesn't matter |  | 0.34 |
| EAT-26 | Particularly avoid food with a high carbohydrate content (i.e. bread, rice, potatoes, etc.) |  | 0.338 |
| STAI-T | I take disappointments so keenly I can't put them out of my mind |  | 0.333 |
| AUDIT | How often during the last year have you found that you were not able to stop drinking once you had started? |  | 0.332 |
| SDS | I have crying spells or feel like it |  | 0.328 |
| BIS | I act on impulse. |  | 0.321 |
| STAI-T | I get in a state of tension or turmoil as I think over my recent concerns and interests |  | 0.318 |
| BIS | I buy things on impulse. |  | 0.315 |
| SSMS | When in the dark do you often see shapes and forms even though there is nothing there? |  | 0.312 |
| SSMS | Are your thoughts sometimes so strong that you can almost hear them? |  | 0.311 |
| BIS | I do things without thinking. |  | 0.31 |
| SSMS | Do you feel that your accidents are caused by mysterious forces? |  | 0.306 |
| SSMS | Do you at times have an urge to do something harmful or shocking? |  | 0.304 |
| SDS | I have trouble with constipation. |  | 0.302 |
| SSMS | Do you often overindulge in alcohol or food? |  | 0.3 |
| AES | Someone has to tell me what to do each day |  | 0.3 |
| SDS | I get tired for no reason. |  | 0.296 |
| STAI-T | I feel nervous and restless |  | 0.295 |
| AUDIT | Have you or someone else been injured because of your drinking? |  | 0.293 |
| BIS | I act on the spur of the moment. |  | 0.293 |
| SSMS | Do ideas and insights sometimes come to you so fast that you cannot express them all? |  | 0.292 |
| SSMS | Does a passing thought ever seem so real it frightens you? |  | 0.292 |
| SSMS | Have you ever thought that you had special, almost magical powers? |  | 0.291 |
| AUDIT | How often do you have six or more drinks on one occasion? |  | 0.286 |
| SSMS | Have you sometimes sensed an evil presence around you, even though you could not see it? |  | 0.281 |
| SDS | I am more irritable than usual. |  | 0.279 |
| AUDIT | How many drinks containing alcohol do you have on a typical day when you are drinking? |  | 0.269 |
| SDS | I feel that others would be better off if I were dead. |  | 0.267 |
| SSMS | Do you ever feel that your speech is difficult to understand because the words are all mixed up and don’t make sense? |  | 0.266 |
| BIS | I spend or charge more than I earn. |  | 0.265 |
| SDS | I notice that I am losing weight. |  | 0.265 |
| LSAS | Eating in public places. |  | 0.265 |
| EAT-26 | Avoid foods with sugar in them |  | 0.263 |
| BIS | I am restless at the theater or lectures. |  | 0.262 |
| SSMS | Would you like other people to be afraid of you? |  | 0.258 |
| SSMS | Do you often feel like doing the opposite of what other people suggest even though you know they are right? |  | 0.257 |
| EAT-26 | Take longer than others to eat my meals. |  | 0.255 |
| SSMS | Do you think that you could learn to read other's minds if you wanted to? |  | 0.251 |
| AES | I approach life with intensity |  | 0.251 |
|  |  |  |  |
|  |  |  |  |
|  |  |  |  |
|  |  |  |  |
|  |  |  |  |

**Summary of item loadings onto ‘Compulsive Behavior and Intrusive Thought’ factor. The top loading items from each questionnaire are displayed in descending order, provided they are above a threshold loading of +/- 0.25.**

**Supplementary File 2C. ‘Top loading items on ‘Social Withdrawal’ factor**

| **Factor 3: Social Withdrawal** | | | |
| --- | --- | --- | --- |
| **Questionnaire** | **Item** |  | **Loading** |
| LSAS | Being the center of attention. |  | 0.769 |
| LSAS | Meeting strangers. |  | 0.765 |
| LSAS | Speaking up at a meeting. |  | 0.751 |
| LSAS | Talking with people you don’t know very well. |  | 0.735 |
| LSAS | Going to a party. |  | 0.699 |
| LSAS | Participating in small groups. |  | 0.677 |
| LSAS | Giving a report to a group. |  | 0.668 |
| LSAS | Expressing a disagreement to people you don’t know very well. |  | 0.636 |
| LSAS | Calling someone you don’t know very well. |  | 0.632 |
| LSAS | Giving a party. |  | 0.624 |
| LSAS | Acting, performing or giving a talk in front of an audience. |  | 0.619 |
| LSAS | Working while being observed. |  | 0.613 |
| LSAS | Entering a room when others are already seated. |  | 0.602 |
| LSAS | Looking at people you don’t know very well in the eyes. |  | 0.576 |
| LSAS | Trying to pick up someone. |  | 0.567 |
| LSAS | Writing while being observed. |  | 0.552 |
| LSAS | Talking to people in authority. |  | 0.527 |
| LSAS | Resisting a high-pressure salesperson. |  | 0.519 |
| SSMS | Do you dread going into a room by yourself where other people have already gathered and are talking? |  | 0.503 |
| LSAS | Returning goods to a store. |  | 0.495 |
| SSMS | Do you (not) like mixing with people? |  | 0.415 |
| LSAS | Taking a test. |  | 0.386 |
| SSMS | Do you prefer watching television to going out with people? |  | 0.378 |
| LSAS | Drinking with others in public places. |  | 0.374 |
| LSAS | Urinating in a public bathroom. |  | 0.35 |
| LSAS | Telephoning in public. |  | 0.342 |
| AUDIT | How ~~often~~ (rarely) do you have six or more drinks on one occasion? |  | 0.327 |
| STAI-T | I lack self-confidence |  | 0.315 |
| LSAS | Eating in public places. |  | 0.305 |
| AES | Getting together with friends is (not) important to me |  | 0.294 |
| BIS | I plan tasks carefully. |  | 0.292 |
| BIS | I (do not) act on the spur of the moment. |  | 0.282 |
| AUDIT | How ~~many~~ (few) drinks containing alcohol do you have on a typical day when you are drinking? |  | 0.278 |
| AUDIT | How ~~often~~ (rarely) during the last year have you been unable to remember what happened the night before because of your drinking? |  | 0.266 |
| AUDIT | How often do you have a drink containing alcohol? |  | 0.260 |
| AES | I (do not) have friends |  | 0.259 |
| BIS | I plan trips well ahead of time. |  | 0.256 |
| BIS | I do (not do) things without thinking. |  | 0.253 |
|  |  |  |  |

**Summary of item loadings onto ‘Social Withdrawal’ factor. The top loading items from each questionnaire are displayed in descending order, provided they are above a threshold loading of +/- 0.25. Words in parentheses, e.g. “(do not)” are added here (but were not presented to participants) to facilitate interpretation of the direction of effects for items that are reverse-coded.**
