# Supplementary material for: Characterizing a psychiatric symptom dimension related to deficits in goal-directed control
Source: eLife. 2016 Mar 1;5:e11305. doi: 10.7554/eLife.11305 (PMC4786435; doi:10.7554/eLife.11305)
Supplement: Supplementary file 3. — Plotted here are regression lines indicating the strength of the relationship between model-based deficits and Factor 2 (‘Compulsive Behavior and Intrusive Thought’) in putative patients (top 25%, blue) and in subjects in the normal range (bottom 75%, red). Each subplot reflects a different subset of the population, based on that clinical questionnaire. Eighteen independent analyses were subsequently carried out (i.e. two per subplot). DOI: http://dx.doi.org/10.7554/eLife.11305.013 [file elife-11305-supp3.docx]

**Supplementary File 3. The relationship between model-based learning and Factor 2 is broadly consistent across ‘putative patients’ (top 25%) and subjects scoring in the normal range (bottom 75%).**

**Plotted here are regression lines indicating the strength of the relationship between model-based deficits and Factor 2 (‘Compulsive Behavior and Intrusive Thought’) in putative patients (top 25%, blue) and in subjects in the normal range (bottom 75%, red). Each subplot reflects a different subset of the population, based on that clinical questionnaire. Eighteen independent analyses were subsequently carried out (i.e. two per subplot).**
