# Supplementary material for: Characterizing a psychiatric symptom dimension related to deficits in goal-directed control
Source: eLife. 2016 Mar 1;5:e11305. doi: 10.7554/eLife.11305 (PMC4786435; doi:10.7554/eLife.11305)
Supplement: Supplementary file 4. — Features that are significantly associated with model-based learning identified using elastic net regularization with tenfold cross-validation, observed in >95% of 100 iterations tested. Index refers to the item number from the questionnaire of origin. Beta refers to the coefficient from the regularized regression model. Words in parentheses, e.g. '(do not)' are added here (but were not presented to participants) to facilitate interpretation of the direction of effects for items that are reverse coded. The last column 'FA Loadings' indicates the significant overlap in terms of loading on Factors F1 (‘Anxious-Depression’), F2 (‘Compulsive Behavior and Intrusive Thought’) or F3 (‘Social Withdrawal), in the positive (+) or negative (-) direction using a cut-off at loadings >= 0.25. DOI: http://dx.doi.org/10.7554/eLife.11305.014 [file elife-11305-supp4.docx]

**Supplementary File 4. Significant Predictors of Goal-Directed (Model-Based) Learning from Supervised Analysis**

| ***Negative Predictors of Goal-Directed Learning Performance*** | | | | | |  |
| --- | --- | --- | --- | --- | --- | --- |
| **Questionnaire** | | **Item** | **Index** | | **Beta** | **FA loadings** |
| Schizotypy | | Can some people make you aware of them by just thinking about you? | SSMS-08 | | -0.025 | - |
| Eating Disorders | | Am preoccupied with the thought of having fat on my body | EAT-14 | | -0.023 | +F2 |
| Impulsivity | | I spend or charge more than I earn | BIS-25 | | -0.020 | +F1; +F2 |
| Schizotypy | | Does a passing thought ever feel so real it frightens you? | SSMS -09 | | -0.019 | +F2 |
| Impulsivity | | I (do not) like to think about complex problems. | BIS-15 | | -0.018 | - |
| Impulsivity | | I do things without thinking | BIS-02 | | -0.017 | +F1; +F2; -F3 |
| Eating Disorders | | Like my stomach to be empty | EAT-24 | | -0.017 | +F2 |
| Impulsivity | | I get easily bored when solving thought problems | BIS-18 | | -0.016 | - |
| Schizotypy | | Do you often feel like doing the opposite of what other people suggest even though you know they are right? | SSMS-43 | | -0.015 | +F2 |
| Depression | | I notice that I am losing weight | SDS-07 | | -0.015 | +F2 |
| Depression | | My heart beats faster than usual | SDS-09 | | -0.015 | +F2 |
| OCD | | I feel that there are good and bad numbers | OCI_16 | | -0.014 | +F2 |
| Alcohol Addiction | | How often during the last year have you been unable to remember what happened the night before because of your drinking? | AUDIT-08 | | -0.014 | +F2; -F3 |
| Eating Disorders | | Am terrified about being overweight | EAT-01 | | -0.014 | +F2 |
| Schizotypy | | When in the dark, do you often see shapes and forms even though there is nothing there? | SSMS-01 | | -0.014 | +F2 |
| OCD | | I check things more often than necessary | OCI-02 | | -0.013 | +F2 |
| Depression | | I (do not) eat as much as I used to | SDS-05 | | -0.013 | - |
| Impulsivity | | I (do not) like puzzles | BIS-29 | | -0.012 | - |
| Apathy | | Someone has to tell me what to do each day | AES-10 | | -0.011 | +F2 |
| Eating Disorders | | I vomit after I have eaten | EAT-09 | | -0.011 | +F2 |
|  | |  |  | |  |  |
| ***Positive Predictors of Goal-Directed Learning Performance*** | | | | |  |  |
| **Questionnaire** | **Item** | | | **Index** | **Beta** | **FA Loadings** |
| Schizotypy | Do you (not) consider yourself to be a pretty much average sort of person? | | | SSMS -34 | 0.032 | - |
| Social Anxiety | Giving a party | | | LSAS-23 | 0.021 | +F3 |
| Apathy | I (do not) approach life with intensity | | | AES-07 | 0.019 | +F1; -F2 |
| Social Anxiety | Calling someone you don’t know very well | | | LSAS-10 | 0.018 | +F3 |
| Schizotypy | Do you often have difficulties in controlling your thoughts | | | SCZ-18 | 0.016 | +F1; +F2 |
| Depression | I (do not) feel that I am useful and needed | | | SDS-17 | 0.015 | +F1 |
| Apathy | I (do not) spend time doing things that interest me | | | AES-9 | 0.012 | +F1 |
| Schizotypy | Are you much too independent to get involved with other people? | | | SCZ-25 | 0.010 | - |
|  |  | | |  |  |  |

**Features that are significantly associated with model-based learning identified using elastic net regularization with tenfold cross-validation, observed in >95% of 100 iterations tested.** **Index refers to the item number from the questionnaire of origin.** **Beta refers to the coefficient from the regularized regression model. Words in parentheses, e.g. “(do not)” are added here (but were not presented to participants) to facilitate interpretation of the direction of effects for items that are reverse coded.**

**The last column “FA Loadings” indicates the significant overlap in terms of loading on Factors F1 (‘Anxious-Depression’), F2 (‘Compulsive Behavior and Intrusive Thought’) or F3 (‘Social Withdrawal), in the positive (+) or negative (-) direction using a cut-off at loadings >= 0.25.**
