# Supplementary material for: Characterizing a psychiatric symptom dimension related to deficits in goal-directed control
Source: eLife. 2016 Mar 1;5:e11305. doi: 10.7554/eLife.11305 (PMC4786435; doi:10.7554/eLife.11305)
Supplement: Supplementary file 5. — *p<0.05 ** p<0.01 ***p<0.001. Each row reflects the results from an independent analysis where each questionnaire total score (z-transformed) was entered as SymptomScorez in the following model: lm(Model-Based-Learning ~ IQz + Agez + Gender + SymptomScorez). Statistics refer to the main effect of SymptomScorez on Model-Based-Learning, i.e. the extent to which that questionnaire total score is associated with changes in model-based learning (which was defined for each participant using the full computational model). (B) Comparing the predictive power of model-based learning defined using the computational model versus one-trial back regression analysis. Each row reflects the results from an independent analysis where each questionnaire total score (z-transformed) was entered as SymptomScorez in the following model: lm(SymptomScorez ~ ModelBasedScore). Prior to conducting these analyses, we regressed out the effects of age, gender and IQ so that we could directly compare the r2 of the models. ModelBasedScore was derived from the one-trial back regression (first three columns) or the computational model (last three columns results). For each, positive β values indicate that the ModelBasedScore is associated with fewer symptoms, whereas negative β values indicate that the symptom score is associated with increased symptoms. The difference between the two approaches is negligible. However the computational model did produce nominally higher r2 and lower p-values for the relationship between clinical scores and model-based learning. DOI: http://dx.doi.org/10.7554/eLife.11305.015 [file elife-11305-supp5.docx]

**Supplementary File 5A. Association between questionnaire total scores and model-based learning defined using full computational model**

| **Construct** | **β (SE)** | ***z*-value** | ***p*-value** |
| --- | --- | --- | --- |
| Experiment 1 |  |  |  |
| OCD | -0.027(0.02) | -1.604 | .109 |
| Depression | -0.010(0.02) | -0.607 | .543 |
| Trait Anxiety | -0.002(0.05) | -0.114 | .910 |
|  |  |  |  |
| Experiment 2 |  |  |  |
| **Impulsivity** | **-0.036(0.01)** | **-3.75** | **<.001 ***** |
| **Eating Disorders** | **-0.035(0.01)** | **-3.597** | **<.001 ***** |
| **OCD** | **-0.028 (0.01)** | **-2.813** | **.005 **** |
| **Alcohol Addiction** | **-0.022(0.01)** | **-2.200** | **.028 *** |
| **Schizotypy** | **-0.020(0.01)** | **-2.020** | **.044 *** |
| Depression | -0.016(0.01) | -1.690 | .091 |
| Trait Anxiety | -0.012(0.03) | -1.224 | .221 |
| Apathy | -0.005(0.01) | -0.548 | .584 |
| Social Anxiety | 0.003(0.01) | 0.286 | .775 |
|  |  |  |  |
| Factors |  |  |  |
| ‘Anxious-Depression’ | -0.007(0.01) | -0.716 | .474 |
| **‘Compulsivity and Related Cognitions’** | **-0.045(0.01)** | **-4.532** | **<.001 ***** |
| ‘Social Withdrawal’ | 0.008(0.01) | 0.773 | .440 |
|  |  |  |  |

****p*<.05 ** *p*<.01 ****p*<.001**

**Each row reflects the results from an independent analysis where each questionnaire total score (z-transformed) was entered as SymptomScorez in the following model: lm(Model-Based-Learning ~ IQz + Agez + Gender + SymptomScorez). Statistics refer to the main effect of SymptomScorez on Model-Based-Learning, i.e. the extent to which that questionnaire total score is associated with changes in model-based learning (which was defined for each participant using the full computational model).**

**Supplementary File 5B. Comparing the predictive power of model-based learning defined using the computational model versus one-trial back regression analysis.**

|  | ***One-Trial Back Regression*** | | | ***Computational model*** | | |
| --- | --- | --- | --- | --- | --- | --- |
| **Clinical Scores** | **β (SE)** | ***p*-value** | ***R^2^*** | **β (SE)** | ***p*-value** | ***R^2^*** |
| Eating Disorders | **-0.09(0.03)** | **<.001***** | **.042** | **-0.09(0.03)** | **<.001***** | **.043** |
| Impulsivity | **-0.09(0.03)** | **.002**** | **.028** | **-0.10(0.03)** | **<.001***** | **.032** |
| OCD | **-0.07(0.03)** | **.012*** | **.050** | **-0.07(0.03)** | **0.005**** | **.051** |
| Alcohol Addiction | **-0.06(0.03)** | **.029*** | **.052** | **-0.06(0.03)** | **.028*** | **.052** |
| Schizotypy | -0.04(0.03) | .101 | .028 | **-0.05(0.03)** | **.044*** | **.029** |
| Depression | -0.03(0.03) | .351 | .031 | -0.05 (0.03) | .09 | .033 |
| Trait Anxiety | -0.02(0.03) | .552 | .038 | -0.03(0.03) | .221 | .038 |
| Apathy | -0.00(0.03) | .897 | .015 | -0.02(0.03) | .584 | .015 |
| Social Anxiety | 0.01(0.03) | .593 | .028 | 0.01(0.03) | .775 | .028 |
|  |  |  |  |  |  |  |
| ‘Anxious-Depression’ | -0.00(0.03) | .967 | .018 | -0.02 (0.03) | .474 | .018 |
| ‘Compulsive Behavior and Intrusive Thought’ | **-0.11(0.03)** | **<.001***** | **.088** | **-0.12(0.03)** | **<.001***** | **.089** |
| ‘Social Withdrawal’ | 0.03(0.03) | .282 | .036 | 0.02(0.03) | .440 | .036 |

**Each row reflects the results from an independent analysis where each questionnaire total score (z-transformed) was entered as SymptomScorez in the following model: lm(SymptomScorez ~ ModelBasedScore). Prior to conducting these analyses, we regressed out the effects of age, gender and IQ so that we could directly compare the r^2^ of the models. ModelBasedScore was derived from the one-trial back regression (first three columns) or the computational model (last three columns results). For each, positive β values indicate that the ModelBasedScore is associated with fewer symptoms, whereas negative β values indicate that the symptom score is associated with increased symptoms. The difference between the two approaches is negligible. However the computational model did produce nominally higher r^2^ and lower *p*-values for the relationship between clinical scores and model-based learning.**
